# Gut Microbial Dysbiosis and Cognitive Impairment in Bipolar Disorder: Current Evidence

**DOI:** 10.3389/fphar.2022.893567

**Published:** 2022-05-23

**Authors:** Wenyu Dai, Jieyu Liu, Yan Qiu, Ziwei Teng, Sujuan Li, Hui Yuan, Jing Huang, Hui Xiang, Hui Tang, Bolun Wang, Jindong Chen, Haishan Wu

**Affiliations:** ^1^ National Clinical Research Center for Mental Disorders, Department of Psychiatry, China National Technology Institute on Mental Disorders, The Second Xiangya Hospital of Central South University, Changsha, China; ^2^ Department of Ultrasound Diagnostic, The Second Xiangya Hospital of Central South University, Changsha, China; ^3^ Department of Radiology, The Second Xiangya Hospital of Central South University, Changsha, China

**Keywords:** bipolar disorder, gut microbiota, cognitive function, gut-brain axis, psychobiotic

## Abstract

Recent studies have reported that the gut microbiota influences mood and cognitive function through the gut-brain axis, which is involved in the pathophysiology of neurocognitive and mental disorders, including Parkinson’s disease, Alzheimer’s disease, and schizophrenia. These disorders have similar pathophysiology to that of cognitive dysfunction in bipolar disorder (BD), including neuroinflammation and dysregulation of various neurotransmitters (i.e., serotonin and dopamine). There is also emerging evidence of alterations in the gut microbial composition of patients with BD, suggesting that gut microbial dysbiosis contributes to disease progression and cognitive impairment in BD. Therefore, microbiota-centered treatment might be an effective adjuvant therapy for BD-related cognitive impairment. Given that studies focusing on connections between the gut microbiota and BD-related cognitive impairment are lagging behind those on other neurocognitive disorders, this review sought to explore the potential mechanisms of how gut microbial dysbiosis affects cognitive function in BD and identify potential microbiota-centered treatment.

## 1 Introduction

Bipolar disorder (BD) is a mood disorder characterized by recurring manic or hypomanic episodes alternating with depressive episodes, which presence increases the risk of multisystem complications, including cognitive impairment and metabolic disorders, seriously affecting the quality of life (Goldstein et al., 2011; Carvalho et al., 2020). Moreover, symptomatic remission constantly precedes the recovery of psychosocial function following a mood episode in BD patients, which is a trend mostly attributed to persistent neurocognitive impairment (Gitlin and Miklowitz, 2017). Thus, therapies focusing on cognitive improvement are greatly significant, considering the well-being and quality of life of BD patients (Bonnín et al., 2019).

Cognitive impairment in BD patients is characterized by defects in neurocognitive areas, including executive function, verbal and visual memory, working memory, attention, and reaction time (Cullen et al., 2016). Tatay-Manteiga et al. (2018) observed neurocognitive dysfunction across all stages of BD. Selective cognitive decline even persists during remission in BD patients (Ott et al., 2021). Cross-sectional evidence also suggests that neurocognition may worsen with chronic comorbidity progression in BD patients (Berk et al., 2017). Compared to patients with schizophrenia (Barch and Sheffield, 2014) or neurodegenerative diseases, such as Alzheimer’s disease (AD) (Simjanoski et al., 2021), BD patients exhibit a similar cognitive profile but with a milder degree of impairment, suggesting that there is overlapping pathophysiology underlying the cognitive impairment across these diseases. Currently, potential mechanisms of cognitive impairment in BD patients require further exploration. Neuroimaging studies have identified certain structural abnormalities associated with cognition, including a reduction in the prefrontal lobe (Abe et al., 2015) and hippocampal volume (Hoseth et al., 2016) and cortical thinning. A reduction in brain volume, especially in the hippocampus, is mostly attributed to excessive glucocorticoid exposure induced by oxidative stress (Alfarez et al., 2008) and abnormal neuroplasticity induced by inflammation and reduced brain-derived neurotrophic factor (BDNF) concentrations (Mondelli et al., 2011). Multiple studies have also demonstrated that significant associations exist between cognitive impairment in BD and inflammation, oxidative stress, and metabolic disorders (Hoseth et al., 2016; Cuperfain et al., 2020).

With the emergence of microbiome research, gut microbiota and gut-brain hormones have also been linked to cognitive impairment in severe mental disorders (Bioque et al., 2021; Misiak et al., 2020). Gut microbiota may modulate the function of the central nervous system, thereby altering behavior and cognition (Table 1), while the brain can activate signaling pathways affecting immune and metabolic function and host behavior—influencing the population and composition of the gut microbiota. The 2-way crosstalk between the central nervous system and gut microbiota *via* various routes including the immune system, enteroendocrine signaling, the vagus nerve and the enteric nervous system, as well as multiple gut microbial metabolites is known as the brain-gut axis (Cryan et al., 2019). The gut microbiota might be involved in postnatal development and have long-term implications for brain function (Sharon et al., 2016). Increased intestinal permeability promotes the release of pro-inflammatory cytokines and microbial-derived metabolites [e.g., lipopolysaccharides (LPS) and lipoproteins] into the circulatory system, causing systemic inflammation and blood-brain barrier impairment (Braniste et al., 2014; Schirmer et al., 2016; Houser and Tansey, 2017; Zhao et al., 2017). Neurochemical signals activated by the gut microbiota can be transmitted from the enteric nervous system to the central nervous system *via* the vagus nerve (Forsythe et al., 2014; Fung et al., 2017). Gut microbial dysbiosis also alters the expression of 5-hydroxytryptamine (5-HT) receptors, neurotrophic factors (e.g., BDNF), and N-methyl-d-aspartic acid (NMDA) receptor subunits in the hippocampus (Bercik et al., 2011), as well as myelin formation in the prefrontal cortex (Hoban et al., 2016a), leading to impaired social cognition. Such extensive overlapping mechanisms suggest a significant role of the gut microbiota in the development of cognitive impairment in BD.

**TABLE 1:** Gut microbial dysbiosis can affect cognitive function in rodent models.

References	Model type	Effects on cognitive function	Mechanism
Bercik et al. (2011)	germ-free mice	reduced exploratory behavior	reduced hippocampal levels of BDNF
Gareau et al. (2011)	germ-free mice	memory dysfunction	gut microbial dysbiosis; dysfunction of HPA axis
Crumeyrolle-Arias et al. (2014)	germ-free mice	deficits in social interaction	decreased dopaminergic turnover rate in the frontal cortex, hippocampus, and striatum
Desbonnet et al. (2014)	germ-free mice	social impairments and decreased social preference	gut microbial dysbiosis; modulation of immune cell cytokines release, changes in vagal nerve activity, and neuroendocrine function
Hoban et al. (2016b)	germ-free mice	anxiety-related behaviors and impaired social cognition	hypermyelinated axons in the prefrontal cortex
Luczynski et al. (2016)	germ-free mice	increased maladaptive stress responsivity	expansion and dendritic morphological changes in the amygdala and hippocampal
Lu et al. (2018)	germ-free mice	deficits in spatial memory, learning memory, and social novelty	abnormal morphological development and maturation in the grey and white matter
Desbonnet et al. (2015)	antibiotic-treated mice	deficits in memory and social interaction	altered dynamic of the tryptophan metabolic pathway; reduced BDNF, oxytocin, and vasopressin expression
Fröhlich et al. (2016)	antibiotic-treated mice	deficits in novel object recognition	gut microbial dysbiosis; brain region-specific changes in the expression of cognition-relevant signaling molecules, notably BDNF, NMDA receptor subunit 2B, serotonin transporter, and neuropeptide Y system
Hoban et al. (2016a)	chronic antibiotic-treated mice	deficits in spatial memory	altered CNS serotonin concentration along with changes in the mRNA levels of corticotrophin-releasing hormone receptor 1 and glucocorticoid receptor
Möhle et al. (2016)	antibiotic-treated mice	decreased working memory	decreased hippocampal neurogenesis; reduced Ly6C(hi) monocytes
Ceylani et al. (2018)	antibiotic-treated mice	decreased locomotor activity and impaired recognition memory	lower levels of serum BDNF are not associated with cognitive impairment but with changes in affective-like behaviors
Zhan et al. (2018)	senescence-accelerated mouse prone 8	deficits in learning and memory of spatial orientation	gut microbial dysbiosis
Lee et al. (2020a)	fecal transplant gavages from aged mice	depressive-like behavior, impaired short-term memory, and impaired spatial memory	decreased fecal SCFAs, acetate, propionate, and butyrate
Lee et al. (2020b)	oral gavage of *Escherichia coli*	deficits in spatial learning and memory	gut microbial dysbiosis; release of lipopolysaccharide; stimulation of vagal-dependent gut-brain signaling
Pearson-Leary et al. (2020)	short-defeat latencies/vulnerable rats	increased depressive-type behaviors	inflammation in the ventral hippocampus; higher microglial density and IL-1β expression in the ventral hippocampus
Xie et al. (2020)	fecal transplant gavages from septic mice	learning impairments and anxiety-like behaviors	gut microbial dysbiosis; stimulation of vagal-dependent gut-brain signaling
Wang et al. (2021)	fecal transplant gavages from sleep deprivation patients in germ-free mice	deficits in attention and memory domain	metabolic dysbiosis; increased neuroinflammation and microglial activity in the hippocampus and medial prefrontal cortex
Hua et al. (2021)	spared nerve injury mice	deficits in spatial learning and memory	the increase of *Actinobacteria*, *Proteus*, and *Bifidobacterium*; disturbances of lipids and amino acid metabolism

**FIGURE 1:**
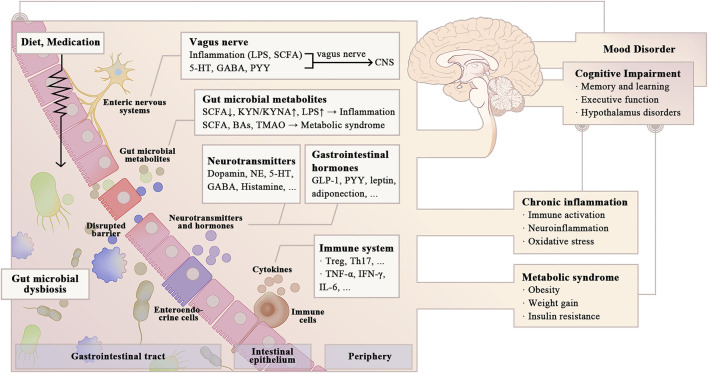
Possible pathways for gut microbial dysbiosis to affect cognitive function in BD. Gut microbial dysbiosis is widely viewed in BD patients, which may have a negative effect on cognitive function (e.g., learning and memory, executive function, and cognitive flexibility). Alterations in the gut microbial composition in BD patients increase intestinal permeability, promoting the release of pro-inflammatory cytokines and microbial-derived metabolites into the circulatory system, causing systemic inflammation and metabolic dysbiosis. Gut microbiota also modulates energy metabolism and cognitive function by influencing the synthesis of neurotransmitters and gastrointestinal hormones, as well as the vagal-dependent gut-brain signaling. Psychiatric medications and changing diet patterns in BD patients have complex interactions with gut microbiota, thereby influencing cognitive function. LPS, lipopolysaccharides; SCFA, single-chain fatty acids; GABA, γ-aminobutyric acid; PYY, Peptide YY; KYN, kynurenine; KYNA, kynurenic acid; Ba, bile acid; TMAO, trimethylamine-N-oxide; NE; GLP-1, glucagon-like peptide-1; Treg, regulatory T cell; Th, helper T cell; INF, Interferon; IL, Interleukin.

Studies explicitly linking the gut microbiota to cognitive impairment in BD remain limited in number compared to those focusing on other neurodegenerative and psychiatric disorders. Therefore, this review summarizes preclinical and clinical evidence to explore potential mechanisms by which the gut microbiota affects cognitive function in BD patients and identify potential microbiota-centered treatment. Studies linking the gut microbiota to cognitive impairment in neurodegenerative and mental disorders might have implications for BD patients. Several studies have addressed the involvement of microbial-derived metabolites [e.g., single-chain fatty acids (SCFAs), secondary bile acids (BAs), and LPS], neurotransmitters, and gastrointestinal hormones in cognitive function. Complex interactions with the gut microbiota may also explain some cognitive side effects of certain psychiatric medications (Figure 1). This review concludes by discussing potential cognitive treatment targeting the gut microbiota, which may improve the quality of life of BD patients.

### 2 Direct Effects of the Gut Microbiota and its Metabolites on Cognitive Function in BD Patients

Evidence of altered gut microbial composition in BD patients suggests that gut microbial dysbiosis contributes to disease progression and pathophysiology in BD patients (Evans et al., 2017; Lai et al., 2020; McIntyre et al., 2021). Although there are few studies discussing the relationship between gut microbiota and cognitive impairment in BD patients, some have found that specific gut microorganisms are associated with reduced cognitive function (Severance et al., 2016). The relationship between the gut microbiota and specific cognitive domains, including learning, memory, attention, processing speed, and executive function—all of which are also impaired in BD—has been demonstrated in animal and human studies on hepatic encephalopathy (Bajaj et al., 2012), diabetes mellitus (Zheng et al., 2021), and aging (Li et al., 2021). Gut microbial dysbiosis also contributes to cognitive impairment in various neurodegenerative and psychiatric disorders, including Parkinson’s disease (Mulak and Bonaz, 2015; Sampson et al., 2016), AD (Jiang et al., 2017), and schizophrenia (Bioque et al., 2021), suggesting potential overlapping mechanisms between these conditions and BD.

### 2.1 Gut Microbial Alterations in BD Patients

**FIGURE 2:**
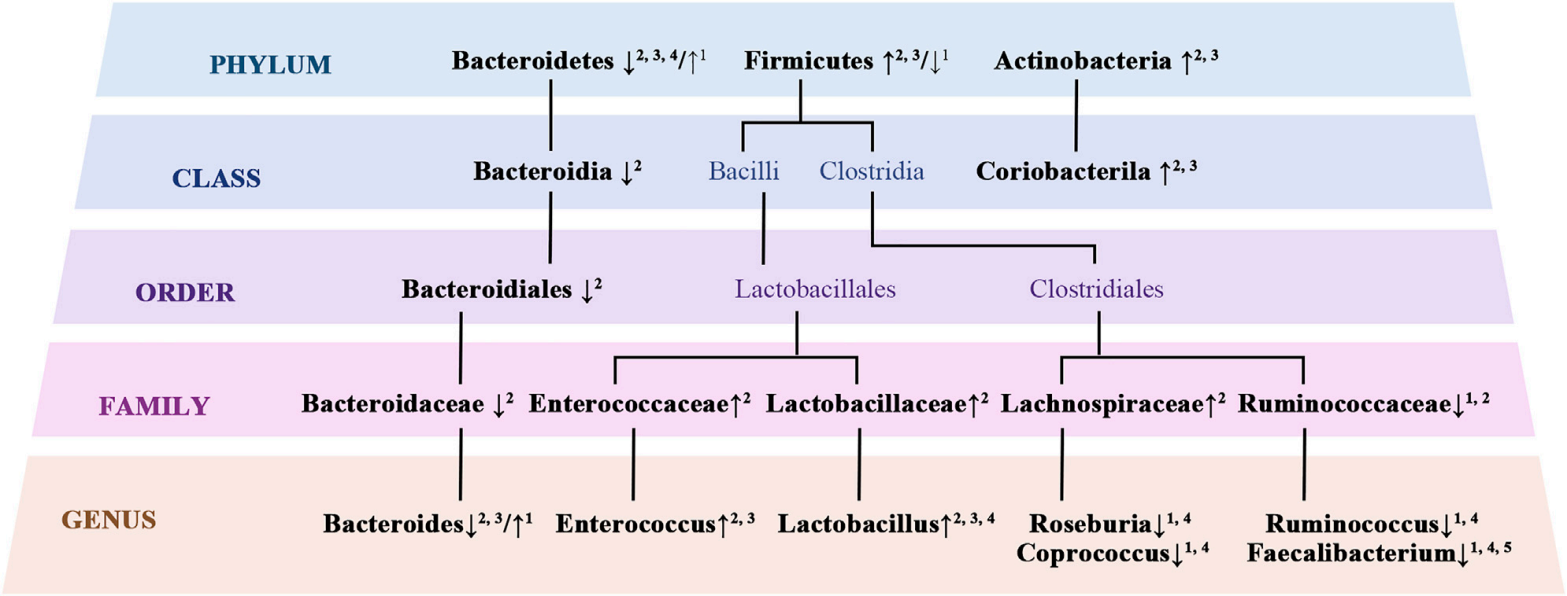
Altered gut microbial composition in BD. 1 (Hu et al., 2019); 2 (Lai J. et al., 2021); 3 (Rong et al., 2019); 4 (Zheng et al., 2020); 5 (Evans et al., 2017)

Disruption of intestinal homeostasis affects the host’s metabolism and immune responses, leading to systemic disorders ranging from metabolic syndrome to chronic inflammation, which is closely related to the development of cognitive impairment (Kesika et al., 2021; Liu et al., 2020; McGrattan et al., 2019; Zeng et al., 2021). Therefore, a quantitative analysis of the gut microbiota in BD patients may help to improve an understanding of the mechanisms behind the development of cognitive impairment in this population. The most dominant gut microbial phyla in healthy adults include Firmicutes, Bacteroidetes, Proteobacteria, Actinobacteria, and Verrucomicrobia, with Firmicutes and Bacteroidetes accounting for nearly 80% of the total amount (Eckburg et al., 2005). In contrast, multiple studies have demonstrated alterations in gut microbial composition in BD patients compared to healthy controls (Figure 2). Lai et al. (2020) found that, in BD patients, counts of *Faecalibacterium prausnitzii*, *Bacteroides*, *Prevotella*, *Atopobium* cluster, *Enterobacter* spp., and *Clostridium* cluster organisms were significantly increased, and the log10 (ratio of *Bifidobacteria* to *Enterobacteriaceae*) was decreased. Another two studies have shown an increase in organisms of the *Firmicutes* and *Bacteroides* phyla but a decrease in those of the *Bacteriodetes* phylum in BD patients, with the Firmicutes-to-Bacteriodetes ratio being accordingly increased (Rong et al., 2019; Lai WT. et al., 2021). An increased Firmicutes-to-Bacteriodetes ratio is a recognized indicator of obesity (Crovesy et al., 2020; Magne et al., 2020). However, bacteria of the *Ruminococcaceae* family and *Faecalibacterium* genus, which are butyrate-producing bacteria, were reduced in BD patients relative to healthy controls in other studies (Hu et al., 2019; Lai WT. et al., 2021; Sublette et al., 2021). In 4 of 5 studies, lower α-diversity was observed in BD patients compared to healthy controls (Sublette et al., 2021).

In conclusion, the most convergent taxonomic finding in BD patients is the reduction in butyrate-producing bacteria, gut microbiota known to impact cognitive function by producing SCFAs, including acetate and butyrate (Tanca et al., 2017). A decrease in butyrate-producing bacteria, such as those of the genus *Faecalibacterium*, was also observed in patients with AD and accompanied by neuroinflammation and impaired cognitive function (Ling et al., 2020; Marizzoni et al., 2020). Associations between cognitive impairment and decreased butyrate-producing bacteria have also been observed in patients with Parkinson’s disease (Nuzum et al., 2020; Tan et al., 2021) and alcohol use disorders (Leclercq et al., 2020) and are likely mediated by butyrate. It has been demonstrated that butyrate levels in the central nervous system can be influenced by gut microbial composition; for example, *Clostridium butyricum*, as a probiotic bacteria, restored butyrate content in the brain and significantly alleviated cognitive impairment and histopathological changes in a mouse model of vascular dementia (Liu et al., 2015). *Clostridium butyricum* treatment also attenuated cognitive impairment and prevented microglia-mediated neuroinflammation in a manner mediated by butyrate in an AD mouse model (Sun et al., 2020). Prolonged treatment with sodium butyrate stimulates neurogenesis and improves memory and associative learning (Kim et al., 2009; Govindarajan et al., 2011). On the one hand, butyrate inhibits histone deacetylase, which plays an important role in intestinal barrier regulation and intestinal energy metabolism, thereby affecting cognitive function (Leonel and Alvarez-Leite, 2012; Tanca et al., 2017). Butyrate can also increase hippocampal neurogenesis and the expression of the neurotrophic factor BDNF, improving learning behavior and long-term memory (Levenson et al., 2004; Sleiman et al., 2016). Considering the neuroprotective and cognitive improvement effects of butyrate, inadequate concentrations of butyrate-producing bacteria may be involved in the pathophysiology of cognitive impairment in BD patients.

### 2.2 Effects of Gut Microbial Metabolites on Cognitive Function in BD Patients

The interaction between butyric acid-producing bacteria and cognitive impairment suggests that microbiota-produced small-molecule metabolites mediate host-microbiome interactions (Donia and Fischbach, 2015). An analysis of brain transcriptomic data from BD patients has also revealed disturbances in gut microbial metabolites, such as tryptophan, SCFAs, and BAs (Moolamalla and Vinod, 2020). These overlapping metabolite profiles suggest potential routes for the involvement of the gut microbiota in cognitive impairment in BD.

#### 2.2.1 Effects of Inflammatory Gut Microbial Metabolites

SCFAs (including acetate, propionate and butyrate), which are typical anti-inflammatory molecules produced by the gut microbiota, can exert anti-inflammatory effects by inhibiting interleukin (IL)-6, IL-1β, and tumor necrosis factor-α (TNF-α) expression through the *FFAR2* (GPR43) receptor (Pirozzi et al., 2018). Animal studies have provided evidence on the effects of SCFAs on cognitive function through immunological pathways. The effects of butyrate on cognitive function have been described above. Additionally, decreased absolute concentrations of acetate, propionic acid, and butyrate lead to blood-brain barrier dysfunction, microglial activation, and elevated cortical IL-1β, IL-6, and TNF-α expression levels in mice maintained on a high-salt diet, exhibiting a reduced number of organisms in the *Bacteroidetes* and *Proteobacteria* phyla and an increased number of those in the *Firmicutes* phylum, respectively (Hu et al., 2020). Mice with a deficiency of the SCFA receptor *FFAR2* showed global defects in microglia similar to those of germ-free mice, suggesting that the gut microbiota regulates microglia maturation and function (Borre et al., 2014; Erny et al., 2015). Microglia cells mediate neuroinflammation as the major innate immune cell population in the brain, playing an important role in the pathophysiology of BD (Maletic and Raison, 2014) and cognitive impairment (Feng et al., 2017; Zhao et al., 2019). However, to our knowledge, studies directly linking SCFAs to cognitive impairment in BD are still scarce. Indirectly, butyrate has been tested as a potential treatment for mood disorders, including BD and major depressive disorder, acting by controlling epigenetic programming associated with cognitive and behavioral regulation as a histone deacetylase inhibitor (Machado-Vieira et al., 2011). Lithium carbonate, one of the most commonly used drugs for treating BD, may activate anti-inflammatory regulatory T-cell responses through an *FFAR2*-dependent mechanism by altering the SCFA-producing gut microbiota (e.g., through upregulation of the butyric acid-producing bacterium *Akkermansia muciniphila*) to change SCFA profiles (Huang et al., 2022). With the support of some clinical studies, given the positive effects of lithium carbonate on cognitive functions, including memory and attention (Dias et al., 2012), disruption of the SCFA profile may impair cognitive function in BD patients through systemic inflammation. Marizzoni et al. (2020) found that both cognitive function and endothelial dysfunction in older adults are positively correlated with the pro-inflammatory cytokines acetate and valerate but negatively correlated with the levels of butyric acid and IL-10. The gut microbiota with reduced SCFA production can also trigger an intestinal inflammatory response and progression of Parkinson’s disease (Unger et al., 2016; Vascellari et al., 2020; Aho et al., 2021). Such clinical evidence suggests that SCFAs are involved in the development of cognitive impairment through systemic inflammation caused by endothelial dysfunction.

**FIGURE 3:**
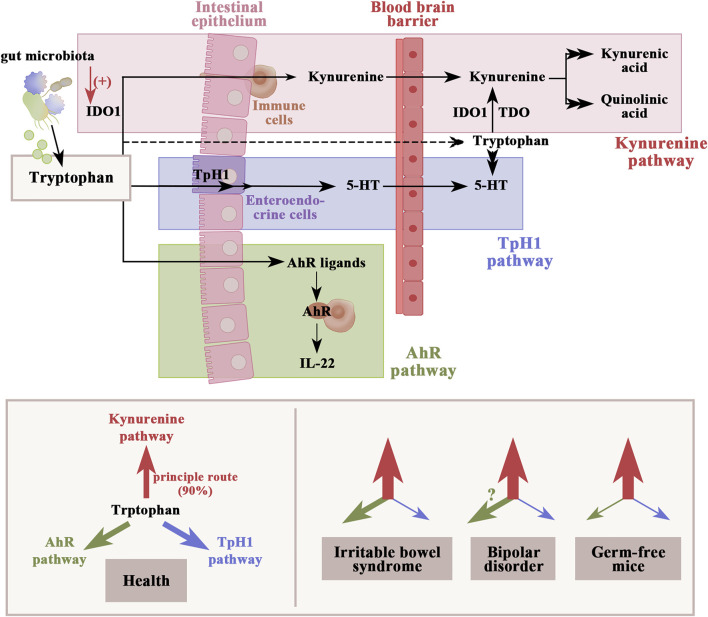
Dietary tryptophan metabolism follows three major pathways in the gastrointestinal tract: 1) the kynurenine pathway *via* IDO1, which can be activated by gut microbial dysbiosis; 2) production of serotonin in enteroendocrine cells and indirect regulation of tryptophan availability under the control of gut microbiota; and 3) the direct conversion to AhR ligands by gut microbiota. The figure shows an altered dynamic of the tryptophan metabolic pathway in IBS, BD, and germ-free mice based on the available clinical data. Weights of arrows indicate the strength of pathway activation. IDO1, indoleamine 2,3-dioxygenase 1; TDO, tryptophan 2,3-dioxygenase; TpH1, tryptophan hydroxylase 1; AhR, aryl hydrocarbon receptor.

Gut-derived tryptophan metabolites are also one of the microbiome-dependent signals regulating inflammatory responses in the host. In the gastrointestinal tract, tryptophan metabolism follows three major pathways, including the kynurenine pathway, the aryl hydrocarbon receptor pathway, and the serotonin production pathway (Figure 3). A review by Więdłocha et al., 2021) supposed that metabolites of tryptophan degradation along the kynurenine pathway not only have an adverse effect on several psychiatric disorders, including BD, schizophrenia, depression, dementia, and AD, but also toxicity on cognitive function. An experiment conducted by Leblhuber et al. (2018) revealed that elevation of the kynurenine pathway may be associated with reduced *Faecalibacterium prausnitzii* and activation of macrophages in AD patients with impaired cognitive function. Notably, BD patients also show reductions in *Faecalibacterium* populations (Hu et al., 2019). Several studies have determined that gut microbial dysbiosis activates indoleamine 2, 3-dioxygenase 1, the rate-limiting enzyme in the kynurenine pathway (Agus et al., 2018). Depletion of the gut microbiota in mice induces elevation of the kynurenine–kynurenic acid pathway, anxiety-like behavior, and cognitive deficits (Desbonnet et al., 2015). The kynurenine–kynurenic acid pathway is also elevated in germ-free mice transplanted with microbiota from schizophrenic patients, leading to impaired learning and memory functions (Zhu et al., 2020). In contrast, performances in both cognitive function (including object exploration and recognition, passive avoidance, and spatial discrimination, all depending on the integrity of hippocampal function) and synaptic plasticity were improved in a rodent model with reduced kynurenic acid synthesis (Potter et al., 2010). Aside from the kynurenine pathway, tryptophan can also be degraded into aryl hydrocarbon receptor agonists by the gut microbiota, which not only protect against increased gut permeability (Scott et al., 2020) but also reduce neuroinflammation by inducing interferon-I signaling in astrocytes (Rothhammer et al., 2016). Therefore, interventions aimed specifically at reducing kynurenine pathway activation may constitute a promising strategy for cognitive improvement in BD patients.

Gut microbial dysbiosis also increases the production of LPS, a pro-inflammatory endotoxin, which diffuses into the blood along with increased gut permeability. LPS also contributes to the development of cognitive impairment through systemic inflammation, as evidenced by lower scores on the Boston naming test measuring visual confrontation naming, Verbal Fluency measuring executive function, and Word List Memory test measuring working memory (Chen et al., 2008). Additionally, LPS has adverse effects on both structure and function in the brain, increasing the activity of the amygdala responsible for emotion control and affecting object-exploration behavior by impairing cognitive function (Haba et al., 2012). LPS also decreases BDNF expression in the hippocampus, inducing cognitive impairment (Dinel et al., 2014). Consistently, gut-derived LPS-related immune activation has been observed in BD patients (Rudzki and Szulc, 2018). In mice transplanted with intestinal flora from BD patients, elevated levels of inflammatory cytokines and *TRANK1* messenger RNA (*TRANK1* is an important risk gene of BD) in the hippocampus and prefrontal cortex may be associated with LPS stimulation of BV-2 microglia (Lai J. et al., 2021). Therefore, LPS leakage due to gut microbial dysbiosis may play an important role in cognitive impairment in BD.

Bidirectional crosstalk between the hypothalamic-pituitary-adrenal (HPA) axis and the gut-brain axis in severe mental illness has been demonstrated in several studies (Misiak et al., 2020; Bioque et al., 2021), in which pro-inflammatory gut microbial metabolites play a key role. SCFAs not only decrease the activity of microglia and limit local inflammatory processes, but also decrease the expression of genes encoding proteins involved in the HPA axis (van de Wouw et al., 2018). LPS can directly stimulate cortisol secretion by human adrenal cells by a cyclooxygenase-dependent mechanism (Vakharia and Hinson, 2005). Reduced SCFA production (Pirozzi et al., 2018) and LPS leakage (Chen et al., 2008) in BD patients can also trigger an intestinal inflammatory response, enhancing release of cytokines, including IL-1β, IL-6 and TNF-α, all of which are potent activators of the HPA axis (Turnbull and Rivier, 1995). Since HPA axis activation and increased basal cortisol are known to be responsible for cognitive impairment including worse performance in visuospatial associative memory, attention and executive function in BD patients (Tournikioti et al., 2018), gut microbial dysbiosis may impair cognitive function in BD patients by activating the HPA axis. A study by Aizawa et al. (2018) provide some insight into the association between gut microbial dysbiosis and the HPA axis dysregulation in BD, as a significant negative correlation between the count of *Bifidobacterium* and cortisol levels was found. Notably, HPA axis dysregulation in BD may in turn further exacerbate gut microbial dysbiosis and intestinal permeability (Vanuytsel et al., 2014; Yoshikawa et al., 2017), thus creating a vicious cycle leading to further cognitive impairment.

#### 2.2.2 Effects of Gut Microbial Metabolites Associated With Metabolic Syndrome

Studies have shown that gut microbial metabolites (including SCFAs, BAs, and LPS) as signaling molecules regulate physiological processes ranging from appetite and intestinal motility to energy metabolism in the host (Heiss and Olofsson, 2018). Therefore, gut microbial dysbiosis may lead to metabolic disorders and contribute to metabolic comorbidities, including diabetes mellitus and insulin resistance, playing an important role in the pathophysiology of various psychiatric diseases, including BD. BD patients presenting with metabolic syndromes are often at high risk of cognitive impairment.

In addition to SCFAs, food-derived gut microbial metabolites include BAs, trimethylamine-N-oxide (TMAO), and glutamate. BAs are synthesized from cholesterol in the liver and further metabolized to secondary bile acids by the gut microbiota (Schmidt et al., 2010). Gut microbial dysbiosis disrupts the signaling of BAs binding to the nuclear farnesoid X receptor (FXR) and Takeda G protein-coupled membrane receptor 5 (TGR5) (Huang et al., 2016; Chiang and Ferrell, 2020), which is also found in the brain (He et al., 2021). Although the effect of BAs on cognitive function in BD remains unclear, an increased ratio of secondary cytotoxic BAs to primary BAs has been linked to AD and cognitive impairment (MahmoudianDehkordi et al., 2019). INT-777, a TGR5 agonist, ameliorates synaptic dysfunction and reverses Aβ1-42-induced cognitive impairment in the mouse model of acute neurotoxicity by upregulating the expression of postsynaptic and presynaptic proteins (PSD95 and synaptophysin) and inhibiting apoptosis (Wu et al., 2018). BAs, such as tauroursodeoxycholic acid, have also been suggested as a potential treatment for AD (Zangerolamo et al., 2021). Thus, BAs may also be potential mediators between gut microbiota and cognitive impairment in BD patients.

TMAO has also been demonstrated to mediate gut microbiota-induced cognitive impairment. Administration of TMAO increases synaptic impairments by inhibiting the mammalian target of the rapamycin signaling pathway and decreases the expression levels of synaptic plasticity-related proteins, thereby exacerbating cognitive impairment (Li et al., 2018). A choline-induced AD mouse model exhibited increased TMAO synthesis, which is positively correlated with cognitive deterioration (Wang et al., 2020). Therefore, the role of TMAO in BD patients deserves further investigation.

## 3 The Role of Gastrointestinal Hormones and Neurotransmitters in the Interaction Between the Gut Microbiota and Cognitive Impairment in BD

The brain–gut axis reflects a complex bidirectional communication network between the gut microbiota and the brain, which relies on various neurotransmitters, gastrointestinal hormones, cytokines, and growth factors (Quigley, 2017; Cryan et al., 2019). These neurotransmitters and gastrointestinal hormones are produced by entero-endocrine cells in response to gut microbial inducement (Heiss and Olofsson, 2018). The gut microbiota also regulates the metabolisms of amino acids essential for the synthesis of neurotransmitters and gastrointestinal hormones (Holzer, 2016). Thus, gastrointestinal hormones and neurotransmitters may be important mediators in the interaction between the gut microbiota and cognitive impairment in BD.

### 3.1 The Role of Gastrointestinal Hormones

Gut microbial dysbiosis increases the intestinal permeability and penetration of pro-inflammatory factors, such as LPS, leading to intestinal inflammation, which, in turn, promotes energy absorption and reduces satiety *via* gastrointestinal hormones (Gomes et al., 2018; Nagpal et al., 2018). These gastrointestinal hormones have receptors expressed in regions of the brain that regulate not only hunger and energy metabolism but also stress, behaviors, and cognitive function (Chan et al., 2003; Bucinskaite et al., 2009).

Glucagon-like peptide-1 (GLP-1), an insulinotropic hormone secreted by entero-endocrine L-cells, can stimulate glucose-dependent insulin secretion in response to carbohydrate uptake (Holt & Trapp, 2016). In rodent models, butyrate and propionate stimulate GLP-1 secretion from entero-endocrine L-cells *via FFAR2* (Tolhurst et al., 2012). Therefore, reduced concentrations of butyrate-producing bacteria in BD patients may downregulate GLP-1 secretion. GLP-1 receptors are expressed in the cerebral cortex, hypothalamus (ventral medial nucleus and arcuate nucleus), and limbic system (amygdala and hippocampus), all of which are regions responsible for the regulation of emotion and cognition (Heppner et al., 2015). GLP-1 and GLP-1 receptor (GLP-1R) signaling have a neuroprotective role in the control of insulin resistance, synaptic plasticity, and neuroinflammation and improve cognitive function in learning, memory, executive function, and attention (Müller et al., 2019; Flintoff et al., 2021). Therefore, reduced GLP-1R signaling caused by reduced butyrate-producing bacteria in BD patients may impair synaptic plasticity and cognitive function, as demonstrated by several studies. Liraglutide, a GLP-1 agonist, reversed manic-like symptoms and impairment in working and recognition memory by improving hippocampal oxidation and BDNF levels in a D-amphetamine-induced BD model (Chaves Filho et al., 2020). Serum GLP-1 levels are significantly lower in BD patients compared to healthy controls and negatively correlate with previous mood episodes (Rosso et al., 2015). In non-diabetic BD patients, liraglutide had beneficial effects on several cognitive domains, including auditory verbal learning, working memory, and attention (Mansur et al., 2017). Therefore, it is likely that gut microbial dysbiosis affects cognitive function in BD patients *via* GLP-1R signaling.

Peptide YY (PYY) is another intestinal satiety hormone produced by entero-endocrine L-cells, which affects the central nervous system by inhibiting orexigenic neurons expressing neuropeptide Y (Wynne and Bloom, 2006). Although there is limited evidence directly linking PYY to BD, a reduction in peripheral PYY concentrations might underlie the lack of GABA inhibition associated with impaired cognitive function in BD, considering that both neuropeptide Y and γ-aminobutyric acid (GABA) are released by arcuate neuropeptide Y neurons (Acuna-Goycolea et al., 2005; Huber et al., 2018).

Adipokines, represented by adiponectin and leptin, are cytokines or hormones secreted by white adipocytes in response to increased circulating inflammatory factors (Aguilar-Valles et al., 2015). Gut microbial dysbiosis elicits the penetration of microbial components (e.g., LPS), compromising leptin signaling and leading to leptin resistance and sustained high leptin levels (Faggioni et al., 1997). Intracerebroventricular administration of LPS induces an elevation in leptin receptors in the hippocampus, accompanied by impaired learning and memory, suggesting that leptin signaling disturbances in the hippocampus are involved in the regulation of cognitive responses (Da Ré et al., 2020). Studies suggest that leptin levels may affect cognitive function in BD patients. The TNF-α antagonist, infliximab, reduces plasma leptin levels in BD patients by modulating soluble tumor necrosis factor receptor 2, contributing to better performance on non-literal memory tasks and increased overall cortical volume, both of which are negatively correlated with the leptin level (Mansur et al., 2020). Adiponectin is the most abundant plasma adipokine that regulates energy expenditure, and it improves insulin sensitivity and fatty acid oxidation as an anti-inflammatory factor (Berg et al., 2002; Kubota et al., 2007). In current studies, adiponectin has been shown to improve cognitive function mainly by regulating insulin sensitivity and inhibiting inflammation (Rizzo et al., 2020). Additionally, it alleviates isoflurane-induced cognitive impairment in aging models by activating the p38-mitogen-activated protein kinase pathway and promoting the proliferation of hippocampal precursor cells (Zhang et al., 2019). Studies on adiponectin levels in BD patients remain controversial since body mass index seems to be more strongly correlated with adiponectin levels than mood status in BD patients (Platzer et al., 2019). Therefore, it is difficult to identify the role of the gut microbiota in the interaction between adiponectin and cognitive function in BD patients, which may be more directly correlated with comorbidities in BD patients.

Cholecystokinin (CCK) is a satiety hormone secreted from I-cells of the proximal small intestines during digestion (Raybould, 2007). Vagal afferent neurons, primary sensory neurons to regulate meal size, exhibit decreased CCK sensitivity when the gastrointestinal tract is colonized with high-fat-type microbiome, leading to increased food intake and body weight (Kim et al., 2020). LPS leakage due to increased inflammatory microbiome including *Bacteroides* and *Prevotella* also impairs CCK-induced satiety, and promotes food intake and excessive weight gain in aging mice (Rubio et al., 2021). Therefore, decreased CCK signaling caused by gut microbial dysbiosis may exacerbate emotional and cognitive impairment associated with obesity and metabolic syndrome. Except for binding to local vagally expressed receptors, periphery CCK can also act on CCKB receptors, which is widely expressed in limbic system (including the hippocampus and the prefrontal cortex), and thus directly affect emotion and cognition (Ballaz et al., 2020). Despite multiple studies showing consistent cognitive enhancing effects by CCKB receptor activation (Taghzouti et al., 1999; Hebb et al., 2005; Plagman et al., 2019), its role is virtually unknown in BD. Only one study by Sears et al. (2013) found associations between suicide attempt and 12 SNPs of CCKB receptors in BD patients. Therefore, further investigation into the role of CCK in cognitive function in BD is warranted.

### 3.2 The Role of Neurotransmitters Regulated by the Gut Microbiota

Gut microbiota can produce most of the neurotransmitters or neuromodulators in the human brain, including dopamine (which can be produced by *Bacillus* and *Serratia*), norepinephrine (produced by *Escherichia*, *Bacillus*, and *Saccharomyces*), serotonin (produced by *Candida*, *Streptococcus*, *Escherichia*, and *Enterococcus*), or GABA (produced by *Lactobacillus* and *Bifidobacterium*) (Donia and Fischbach, 2015; Strandwitz, 2018). These neurotransmitters mediate gut-brain signals, communicating with various regions of the brain, including the frontal cortex, limbic system, and autonomic and neuroendocrine centers, regulating not only appetite-related sensations but also emotion, cognition, and behavior (Holzer, 2016).

Dysregulation of the dopaminergic system contributes to both pathophysiology and impaired executive function in BD (Bercik et al., 2011; Kao et al., 2018). Various gut microbiota can produce dopamine, leading to dopaminergic aberrations when gut microbial dysbiosis occurs in BD (Tsavkelova et al., 2000). Mice treated with the dopamine transporter protein inhibitor GBR12909 (15 mg/kg) constitute a validated animal model for BD, whereas germ-free mice are less susceptible to GBR12909 with less mania-like behavior, suggesting that the gut microbiota contributes to the disease progression of BD *via* the dopaminergic system (de Miranda et al., 2020). Additionally, para-Cresol-treated mice with social-behavioral deficits have a similar gut microbial profile to that of BD patients (e.g., depleted *Clostridiales*), with a reduction in excitability and number of evoked action potentials of dopamine neurons in the ventral tegmental area (Bermudez-Martin et al., 2021). The increase in *Bacteroides* and *Prevotella*, which is also observed in BD patients, is negatively associated with dopamine transporter expression in the brain (Hartstra et al., 2020). The application of probiotics has been demonstrated to prevent cognitive impairment by regulating the gut microbiota, thereby increasing serotonin, dopamine, and GABA levels and restoring neuronal impairment (Song et al., 2021). The evidence presented above strongly supports the interaction between the dopaminergic system and gut microbiota, underlying the cognitive impairment in BD.

Serotonin, also known as 5-HT, is significantly correlated with cognitive function in BD patients (Chou et al., 2012). In clinical studies, tryptophan levels and the kynurenine-dependent tryptophan index were reduced in patients with bipolar mania and were positively correlated with the Young Mania Rating Scale and Brief Psychiatric Rating Scale scores (Myint et al., 2007). Reduced 5-HT levels in the brain lead to impaired cognitive flexibility, which is the characteristic type of cognitive impairment in BD (Evers et al., 2007). Notably, the gut microbiota can divert tryptophan metabolism to the production of kynurenine instead of 5-HT by activating indoleamine 2, 3-dioxygenase and tryptophan 2,3-dioxygenase (Badawy, 2017). A lack of aryl hydrocarbon receptor ligands in the intestinal contents of germ-free mice has been consistently observed (Lamas et al., 2016). In germ-free mice, there are also relatively low plasma kynurenine levels accompanied by increased indoleamine 2,3-dioxygenase activity, and kynurenine levels increase in these animals after recolonization of the gut microbiota (Clarke et al., 2013; Van der Leek et al., 2017). The altered dynamic of the tryptophan metabolic pathway has also been implicated in a study of postmortem anterior cingulate gyrus in BD patients, showing increased tryptophan 2,3-dioxygenase activity and kynurenine levels (Miller et al., 2006). Thus, the activation of the kynurenine pathway by gut microbiota dysbiosis consequently increases tryptophan consumption, which potentially contributes to the reduced 5-HT neurotransmission in BD patients, a statement being supported by studies focusing on comorbid irritable bowel syndrome (IBS) in BD patients (Tseng et al., 2016) (Figure 3). IBS, characterized by abdominal pain, bowel movement disorders, and gut microbial dysbiosis, is considered to be a valid contributor to BD (Castellini et al., 2016; Rosenblat et al., 2020). A follow-up study showed that IBS patients in clinical remission still experience persistent attention impairment, with a continuous increase in plasma IL-6 levels, the kynurenine-to-tryptophan ratio, and inactivation of the cortisol awakening response (Clarke et al., 2020). Acute tryptophan depletion significantly reduces plasma tryptophan and 5-hydroxyindoleacetic acid in IBS patients, inducing a negative shift in affective memory but without significant changes in mood (Kilkens et al., 2004). The evidence above suggests that 5-HT regulation by the gut microbiota may be involved in cognitive impairment in BD patients.

The gut microbiota profoundly affects peripheral GABA levels. Multiple organisms of gut microbiota are also involved in GABA synthesis, including a range of *Bifidobacterium*, *Lactobacillus*, and 16 intestinal *Bacteroides* strains (Barrett et al., 2012; Luck et al., 2021; Otaru et al., 2021). In germ-free animals, there is a significant reduction in GABA levels in the gut, serum, and brain (Matsumoto et al., 2013). Intestinal and peripheral GABA levels may influence the central nervous system through the gastrointestinal vagal nervous system. Bravo et al. (2011) found that treatment with *Lactobacillus rhamnosus* increases GABA(B1b) messenger RNA expression in cortical (cingulate and prefrontal) regions and decreases expression in the hippocampus and amygdala, consequently reducing corticosterone levels and manic-like behaviors. Moreover, the neurochemical and behavioral effects disappeared in vagotomized mice. *Lactobacillus casei* also stimulates gastrointestinal afferent vagal activity and inhibits stress-induced activation of cells producing pro-adrenocorticotropic hormone in the hypothalamic paraventricular nucleus in a dose-dependent manner, ameliorating somatic symptoms induced by learning stress (Takada et al., 2016). As reviewed by Wagner-Skacel et al. (2020), *Lactobacillus* has been significantly associated with the circadian rhythm in BD patients, linking a GABA disorder caused by the gut microbiota to cognitive function in BD. Thus, the gut microbiota may affect the GABA system mostly through the intestinal vagus nerve and modulate stress-related behavioral and cognitive functions in BD patients.

Multiple studies have demonstrated the versatility of glutamate, the agonist of N-methyl-d-aspartate receptor (NMDAR), as the foremost excitatory neurotransmitter in the central nervous system and modulator of gastrointestinal metabolism (Baj et al., 2019). A systematic review conducted by Reddy-Thootkur et al. (2020) shows that hippocampal glutamate levels are increased in BD patients, but no associations between glutamate metabolite levels and memory performance are detected. Considering that many of those included studies suffered from small sample sizes, the relationship between glutamate and cognitive impairment in BD require further exploration (Reddy-Thootkur et al., 2020). A postmortem study in 10 BD patients revealed significant lower protein and mRNA levels of NMDAR, indicating the presence of excitotoxicity induced by abnormal glutamatergic signaling in BD frontal cortex (Rao et al., 2010). Although there is evidence in support of the impact of the glutamatergic system in cognitive decline and disease progression in BD patients, information regarding the possible ways glutamate (either from dietary sources or microbial activities) may influence cognitive function in BD are still scarce. A Pilot study noted that the plasma and fecal glutamate levels, influenced by relative abundance of certain bacterial families, are negatively associated with cognitive function including processing speed, mental flexibility and executive function (Palomo-Buitrago et al., 2019). Dietary or luminal glutamate may also activate vagal afferents which directly or indirectly influence brain areas including the cerebral cortex, limbic system, hypothalamus and basal ganglia (Kondoh et al., 2009). From the foregoing, further study is needed to investigate the impact of a dietary glutamate and gut-endogenous glutamate in cognitive function in BD.

As an important part of monoamine metabolism, the role of D-amino acids in the gut-brain axis is gaining wider attention. Gut microbiota contributes to the host pool of D-amino acids *via* intrinsic amino acid racemases within certain gram-negative microbiome (Radkov and Moe, 2014). Kawase et al. (2017) found that gut microbiota could modulate the metabolism of D-amino acids in the brain. They noted that D-aspartic acid, D-serine were higher in some brain regions of GF mice than in those of SPF mice, indicating that gut microbiota may regulate the activity of aspartic acid racemase and serine racemase in the host brain (Kawase et al., 2017). D-serine is an endogenous ligand for NMDAR and thus play a key role in synaptic plasticity (Schell, 2004). Long-term potentiation, which underlies learning and memory, depends on calcium dependent release of D-serine from astrocytes in adult rat CA1 pyramidal hippocampus cells (Henneberger et al., 2010). Genetic association studies convince the role of D-serine in the pathology of BD, showing an association between BD with the gene *G72*, whose product activates the D-serine degrading enzyme (Chen et al., 2004; Müller et al., 2011). Furthermore, ketamine metabolites (rac)-dehydronorketamine and (2S,6S)-hydroxynorketamine decrease intracellular D-serine concentrations in a concentration dependent manner in PC-12 cells (Singh et al., 2016). Previous study has found potential pro-cognitive effects with intravenous subanesthetic ketamine in BD patients (Zhou et al., 2021). Based on these findings, we suggest that gut microbiota may influence synaptic D-serine availability and thus modulate cognitive function in BD patients.

BDNF can also promote the growth and development of neurons as a neurotrophic factor. Alterations in a 5-HT receptor, BDNF, and NMDA receptor subunit expression in the hippocampal region have been demonstrated in germ-free mice (Bercik et al., 2011). However, studies of BDNF levels and specific functional and behavioral alterations in germ-free or antibiotic-treated animal models remain controversial (Holzer, 2016).

## 4 Interactions Between the Gut Microbiota and Psychiatric Medication

Significantly more attention is being given to the interactions between psychiatric drugs commonly used in BD and the gut microbiota. Cognitive side effects induced using antipsychotics in the treatment of BD include impairments in verbal learning, memory, cognitive control, and spatial working memory (Arts et al., 2013; Flowers et al., 2016). Cognitive side effects have also been previously implicated in chronic inflammation and metabolic syndrome (Fang et al., 2019). Meanwhile, recent studies have considered the interaction between psychiatric medication and gut microbiota as a potential way by which the gut microbiota affects cognitive function (Flowers et al., 2019).

On the one hand, psychiatric medication has an important influence on the gut microbial profile, which, in turn, induces potential adverse events. Valproate significantly reduces fecal microbial richness and induces a gut microbial profile similar to that of patients with autism spectrum disorders (Liu et al., 2018). Flowers et al. (2017) showed that treatment with an atypical antipsychotic (AAP) in BD patients results in reduced gut microbial diversity, especially in women. A subsequent study focusing on patients with BD or schizophrenia also revealed significantly lower gut microbial diversity in AAP users compared to non-AAP users (Flowers et al., 2019). In contrast, Hu et al. (2019) found no significant changes in gut microbial α-diversity but noted an altered gut microbial composition in BD patients treated with AAP monotherapy (quetiapine). After such treatment, organisms of the *Klebsiella* and *Veillonella* genera are significantly increased in BD patients. Moreover, almost all AAPs can lead to weight gain, which may be closely related to gut microbial dysbiosis (McEvoy et al., 2005). Bahr et al. (2015b) found that the administration of risperidone inhibited non-aerobic resting metabolism in the gut microbiota, leading to a reduced total resting metabolism rate and increased body weight. The decreased Bacteroidetes-to-Firmicutes ratio induced by risperidone was linked to secondary weight gain in adolescent children (Bahr et al., 2015a). Zeng et al. (2021) also reviewed the potential role of the gut microbiota in cognitive impairment due to the metabolic side effects of AAPs used as a treatment in schizophrenic patients, which included disruption of inflammatory cytokine signaling and neurotransmitter disorders. Therefore, AAPs are likely to increase the risk of metabolic disorders by affecting the gut microbiota, consequently resulting in cognitive side effects.

On the other hand, the gut microbiota may also help to mediate the cognitive improvement effects of psychiatric medications for BD. Oral selective serotonin reuptake inhibitors (SSRIs) increase the excitability of the intestinal vagal nerve system through an intestinal epithelium-dependent mechanism. Critically, blocking the intestinal vagal signal by subdiaphragmatic vagotomy abolishes the antidepressant effect of oral SSRI treatment (McVey Neufeld et al., 2019). Both behavioral and neuroimaging studies have confirmed the positive effects of SSRI treatment on attention, appraisal, and memory before symptomatic remission. Additionally, Harmer and Cowen (2013) suggested that the antidepressant effects of SSRIs are cumulative results of improvements in cognitive functions related to emotion processing. Accumulated evidence highlights the potential role of vagal-dependent gut-brain signaling in cognitive improvement by SSRI treatment. Aripiprazole treatment also significantly increases the richness and diversity of the gut microbiota, especially the relative abundance of organisms of the minor genera *Clostridium*, *Peptoclostridium*, *Intestinibacter*, and *Christenellaceae*, also accompanied by a rise in acetate, butyrate and isovalerate levels (Cussotto et al., 2019). Increased numbers of butyrate-producing bacteria may underlie the cognitive improvement of aripiprazole (Ning et al., 2021; Peitl et al., 2021). Studies targeting the *in vivo* interactions of various BD medications with both the gut microbiota and cognitive function will provide new insights into the mechanisms and side effects of these drugs. Microbiota-centered treatment will also be important for optimizing the management of BD patients.

## 5 Potential Microbiota-Centered Treatments for Cognitive Improvement

Accumulated understanding of the brain-gut axis has led to the development of microbiota-centered treatment in mental illness acting through the gut flora. Both changes in diet habits and psychobiotic supplements can easily modify the gut microbiota. Fecal microbiota transplantation (FMT) facilitates a more stable evolution of gut microbial transplantation.

### 5.1 Diet

Changing one’s dietary habits is the easiest method to modulate gut microbiota. Gut microbial dysbiosis is a key factor of cognitive impairment in diet-induced obesity (Desbonnet et al., 2014). A dietary survey of 97 BD patients showed that BD patients had greater intake levels of processed meat and sugar, fat, and salt (Davison and Kaplan, 2012). Long-term consumption of a high-fat diet increases the Firmicutes-to-Bacteroides ratio, which is associated with obesity (Shi et al., 2015; Ussar et al., 2015). A high-fat diet also led to insulin resistance and hyperglycemia in diet-induced obese mice who exhibited neurotransmitter disorders, including increased GABA and decreased tryptophan levels (Scott et al., 2020). Further, a high-fat diet can cause a significant decrease in tyrosine phosphorylation of insulin receptors, accompanied by an increase in inflammatory response signals (e.g., nuclear factor kappa-light-chain-enhancer of activated B-cells, c-Jun N-terminal kinase) in whole-brain lysate and a decrease in synaptic plasticity, leading to learning and memory impairment (Kothari et al., 2017).

In contrast, healthy diets have shown therapeutic potential for neurocognitive disorders. The Mediterranean diet, characterized by a high intake of fruits, vegetables, nuts, whole grains, and high-protein foods (i.e., fish), can help to reduce intestinal inflammation, cognitive impairment, and the risk of dementia (Pistollato et al., 2018). People at high risk of cardiovascular diseases also showed higher scores on the mean Mini-Mental State Examination and Cognitive Dysfunction Test after long-term consumption of the Mediterranean diet (Martínez-Lapiscina et al., 2013). In terms of dietary composition, a diet high in protein and saturated fat contributed to a greater abundance of *Bacteroides* (Wu et al., 2011). Fruits and vegetables are rich in dietary fiber. Microbiota-accessible carbohydrates found in dietary fiber increase the richness and α-diversity of the gut microbiota and inhibit the hippocampal glial activation and neuroinflammation induced by a high-fat diet. In turn, these carbohydrates improved the performance of mice in nest-building and temporal order memory tests (Shi et al., 2020). Perez (2018) found that BD patients have lower compliance with the Mediterranean diet and higher biomarkers of insulin resistance compared to the healthy population. Therefore, dietary management of BD patients is crucial for reducing the risk of metabolic disorders and cognitive impairment in clinical practice.

The ketogenic diet is another dietary pattern that has attracted increasing attention in the treatment of neuropsychiatric diseases. Several studies have confirmed the role of the ketogenic diet in improving comorbidities and cognitive function in BD patients. The ketogenic diet reduces hyperinsulinemia in BD patients by alleviating mitochondrial dysfunction mediated by impairment of the phosphatidylinositol-3 kinase/protein kinase B/hypoxia-induced factor-1α signaling pathway (Campbell and Campbell, 2020). The ketogenic diet may affect the function of vesicular glutamate transporters and EAAT, Na+, K + -ATPase, Kir4.1, aquaporin-4, Cx34, and KATP channels by affecting the glutamate-glutamine cycle and glutamate synthase activity in astrocytes, thus reducing mild cognitive impairment in BD patients (Morris et al., 2020). Several studies have confirmed the role of the gut microbiota in the ketogenic diet, although only a few studies have discussed whether the gut microbiota is involved in the cognitive improvement effect of the ketogenic diet in BD patients. The ketogenic diet protects against seizures by regulating gut microbiota colonization, which increases GABA and glutamate levels in the hippocampus (Olson et al., 2018). In AD patients with mild cognitive impairment, a 6-week ketogenic diet increased the abundance of *Enterobacteriaceae*, *Akkermansia*, *Slackia*, *Christensenellaceae*, and *Erysipelotriaceae* and the synthesis of butyrate, accompanied by an improvement in cognitive function (Nagpal et al., 2020).

### 5.2 Psychobiotics

Probiotics are live microorganisms that offer non-specific benefits to the health of the host. Some of them may also modulate functions of the central nervous system, reducing psychiatric symptoms and improving cognitive function in patients, and are therefore known as psychobiotics (Evrensel et al., 2019). Clinical studies have demonstrated the role of probiotics in promoting gut microbial diversity and improving cognitive function in BD. Probiotics, including *Lactobacillus rhamnosus* strain GG and *Bifidobacterium animalis* subsp. lactis strain Bb12, reduce the risk of re-hospitalization among BD patients (Dickerson et al., 2018). A cohort study conducted by Reininghaus et al. (2018) showed a significant improvement in attention and psychomotor processing speed on the Digit Symbol Test and executive function on the Trail Making Test B in BD patients after long-term administration of a probiotic supplement, mainly containing organisms of the *Lactobacillus* genera and *Bifidobacterium* genera.

Because some prebiotics support the growth of specific gut microbiota with psychophysiological effects, some have been designated as psychobiotics, including fructooligosaccharides, inulin, and galactooligosaccharides (Sarkar et al., 2016). Currently, there are limited studies addressing the effect of prebiotics in BD patients, but studies in animal models confirm their potential for cognitive improvement. Prebiotics might suppress inflammation that affects cognitive function. Chitosan oligosaccharides effectively reduced learning and memory impairment in an AD model by inhibiting oxidative stress and reducing the release of pro-inflammatory factors, such as IL-1 and TNF-α (Jia et al., 2016). Prebiotics also regulate the synthesis of gastrointestinal hormones. Four weeks of supplementation with prebiotics increased the expression of anorexigenic gastrointestinal hormones, such as peptide tyrosine-tyrosine, GLP-1, and leptin, while decreasing levels of ghrelin and other anorexigenic hormones and helping to improve learning and memory function in schizophrenia patients (Kao et al., 2018). Weight gain induced by antipsychotics, including olanzapine and risperidone, can also be reduced by prebiotics. Intake of the prebiotic galactooligosaccharide mixture significantly reduced olanzapine-induced weight gain, possibly in association with an increased and decreased number of organisms belonging to the *Bifidobacterium* and *Firmicutes* genera, respectively, together with increased cortical phospho-NMDA receptor 1 levels and decreased plasma TNF-α levels. Therefore, these effects suggest that prebiotics may prevent the metabolic and cognitive side effects of olanzapine.

### 5.3 Gastrointestinal Hormone Analogs

The possible mechanisms by which the gut microbiota modulates energy metabolism and cognitive function by influencing gastrointestinal hormones have been reviewed above. Gastrointestinal hormone analogs may also improve cognitive function in a mode similar to that of gastrointestinal hormones. Liraglutide, the GLP-1 receptor agonist, improved D-amphetamine-induced mania-like symptoms and working and recognition memory impairment in a BD mouse model (Chaves Filho et al., 2020). In non-diabetic BD patients, liraglutide also showed beneficial effects in several cognitive domains, including auditory verbal learning, working memory, and attention (Mansur et al., 2017). Therefore, the neuroprotective effect of liraglutide illustrates the potential for gastrointestinal hormone analogs to serve as promising adjunctive tools for BD treatment.

### 5.4 Fecal Microbiota Transplantation

FMT treatment involves the injection of filtrate feces from a healthy donor into a patient. FMT not only increases the microbial diversity but also provides long-term implantation of donor strains compared to the short-term impact on the gut microbiota achieved by changes in the diet or the addition of psychobiotics (Weingarden and Vaughn, 2017). In animal research, FMT treatment reversed cognitive impairment in AD model mice by altering the gut microbial composition and SCFA profile and increasing synaptic plasticity. Currently, few clinical studies have focused on the feasibility and efficacy of FMT in BD patients, although Hinton (2020) claimed that depressive symptoms disappeared with weight loss in a BD patient who had experienced 9 sessions of FMT treatment. Therefore, FMT may have a potential role in improving the cognitive function of BD patients, but further clinical experiments are warranted.

### 5.5 Vagus Nerve Stimulation

The vagus nerve establishes one of the important connections between emotional and cognitive areas of the brain and gut functions. Vagal afferent fibers express receptors for multiple gastrointestinal hormones (GLP-1, CCK, peptide YY, ghrelin, etc.), neurotransmitters (dopamine, GABA, NE, 5-HT, etc.), and gut microbial metabolites (SCFAs, LPS, etc.), to transfer microbiota signals to the central nervous system (Bonaz et al., 2018; Breit et al., 2018). Brain pathway activated by oral administration of Campylobacter jejuni, which has been proved to influence behavior and brain functions at subclinical doses, has been mapped using c-fos expression as a marker of neuronal activation (Gaykema et al., 2004). In this study, brain activation was observed in the nucleus tractus solitarius, the vagal afferent ending, and the projections of the nucleus tractus solitarius including parabrachial nucleus, paraventricular nucleus of the hypothalamus, amygdala and thalamus, indicating vagally-mediated microbiota effect on mood and cognitive function (Gaykema et al., 2004). Consistently, Bravo et al. (2011) found that treatment with *Lactobacillus rhamnosus* decreases GABA(B1b) messenger RNA expression in the hippocampus and amygdala, consequently reducing corticosterone levels and manic-like behaviors. Moreover, the neurochemical and behavioral effects disappeared in vagotomized mice, indicating therapeutic potential for treatment targeting vagal tone.

Vagus nerve stimulation, a medical treatment that is routinely used in the treatment of epilepsy and other neurological conditions, works by applying electrical impulses to the vagus nerve (Wheless et al., 2018; González et al., 2019). A 5-year prospective research in patients with treatment-resistant bipolar depression showed that treatment with vagus nerve stimulation was associated with better medication response and significantly greater mean reduction in suicidality compared to treatment-as-usual (McAllister-Williams et al., 2020). Chronic vagus nerve stimulation also produces sustained clinical and cognitive improvements in BD patients in a treatment-resistant depressive episode (McAllister-Williams et al., 2020). Therefore, vagus nerve stimulation seems to be a promising adjunctive therapy for cognitive impairment in BD patients.

## 6 Conclusion

There is an exciting future potential for research on the connection between gut microbiota and neurocognitive elements in BD patients. Accumulated studies have offered convincing evidence of the participation of microbial-derived metabolites, neurotransmitters, and gastrointestinal hormones in cognitive function. Complex interactions with the gut microbiota may also explain some of the cognitive side effects of certain psychiatric medications. Understanding the potential mechanisms underlying the gut microbiota and cognitive impairment in BD can unlock the door for the application of microbiota-centered treatments in BD management, which may help to prevent adverse events and improve the quality of life in BD patients. However, further investigation is needed before applying these findings in clinical practice despite applauding the recent rise in these strategies.
